# Comparison of Adductor Canal Block and Femoral Nerve Block for Postoperative Pain in Total Knee Arthroplasty: A Systematic Review and Meta-analysis

**DOI:** 10.1097/MD.0000000000002983

**Published:** 2016-03-25

**Authors:** Cui-Cui Dong, Shu-Ling Dong, Fu-Cheng He

**Affiliations:** From the Department of Clinical Laboratory (C-CD, S-LD, F-CH), The First Affiliated Hospital of Zhengzhou University, Henan Key Laboratory of Medical Laboratory (F-CH), Zhengzhou, Henan, 450000, China.

## Abstract

A total knee arthroplasty (TKA) has always been associated with moderate-to-severe pain. A systematic review of randomized controlled trials (RCTs) and non-RCTs was performed to evaluate the efficacy and safety of pain control of adductor canal block (ACB) and femoral nerve block (FNB) after TKA.

Relevant literatures about the ACB and FNB after TKA for reducing pain were searched from Medline (1996-January, 2015), Embase (1980-January, 2015), PubMed (1980-January, 2015), Web of Science (1980-January, 2015), and The Cochrane Central Register of Controlled Trials. High-quality RCTs and non-RCTs were picked to evaluate the visual analogue scale (VAS) and other outcome. This systematic review and meta-analysis were performed according to the PRISMA statement criteria. The software RevMan 5.30 was used for the meta-analysis.

Eight literatures fitted into the inclusion criteria. There were no significant differences in VAS score with rest or mobilization at 4, 24, and 48 h between ACB group and FNB group. There were also no significant differences in the strength of quadriceps and adductor, the length of hospital stay, and complications of vomiting and nausea.

Present meta-analysis indicated that ACB shows no superiority than FNB group. Both of them can reduce the pain score after TKA. As referred to which method to adopt, it is determined by the preference of the surgeons and anesthesiologists.

## INTRODUCTION

Total knee arthroplasty (TKA) is associated with relatively severe pain and difficult to manage. It has demonstrated that about 60% of patients have severe pain and 30% of patients have moderate pain post-TKA.^1^ The pain after TKA does not only impose restriction on early mobilization but also increases the rates of immobility-related complications such as deep venous thrombosis (DVT). Effective analgesia post-TKA is of extreme importance to the postoperative patients, which can improve the patients’ satisfaction. To relieve the pain and improve the effect of TKA, the most common analgesic methods are patient-controlled intravenous analgesia (PCIA), epidural analgesia, continuous femoral nerve block (FNB).^2,3^

However, PCIA needs a large amount of opioids and is relevant to more adverse events than FNB, and patients who received epidural analgesia had a higher rate of hypotension and urinary retention.^4^ FNB may weaken the strength of quadriceps and thus increase the incidence of the falling.^5,6^ With the advent and development of ultrasonography, the adductor canal as an aponeurotic structure in the middle third of the thigh can be seen clearly. Through this new technology, adductor canal block (ACB) can be successfully implemented and thus can be performed to the knee surgery to relieve the pain. This method selectively blocks the sensory nerve but does not block the motor neuron. So this can relieve pain, meanwhile it does not weaken the strength of quadriceps and adductor, thus reducing the incidence of falling.^7^

Both ACB and FNB can relieve the postoperative pain. However, there is no consensus about which is comparable effective way to relieve the pain after TKA. Based on the present study about the amount about comparison of ACB versus FNB is limited. Thus, we carried out a meta-analysis to improve the evidence to understand whether there were any differences between ACB and FNB in terms of efficacy of the alleviation of pain, the strength of quadriceps and adductor, ambition ability, complication after the ACB and FNB, and the length of hospital stay (LOS).

## MATERIALS AND METHODS

Ethical approval for this study was unnecessary because it was a review of existing literature and did not involve any handling of individual patient data.

### Search Strategy

**FIGURE 1:**
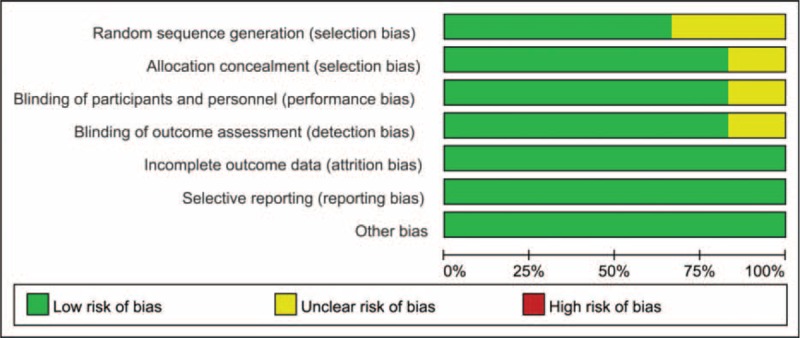
The risk of bias graph.

The electronic databases including Medline, Embase, PubMed, CENTRAL (Cochrane Controlled Trials Register) Web of Science, and Google database were searched for relevant studies involving ACB and FNB in the management of pain relief after TKA in January 2015. The key words and its medical subject heading (Mesh) terms “adductor canal block” “femoral nerve block” “total knee arthroplaty” “total knee replacement” “TKA” “TKR” were combined with Boolean operators AND or OR. The search strategy was presented in Figure 1. Furthermore, the reference lists of all the full-text literatures were reviewed to identify any initially omitted studies and no restrictions on the language of the publication. And the duplicates were excluded by the software of Endnote X7.

### Eligibility Criteria and Study Quality

Study selection was performed according to the following inclusive criteria: patients underwent the primary TKA; intervention including ACB and FNB; the primary outcomes included pain score with rest and mobilization, maximum voluntary isometric contraction (MVIC) of quadriceps and adductor, Timed-Up-and-Go (TUG) test, secondary outcomes are LOS and the complications (vomiting and nausea); the type of studies are randomized control trials (RCTs) and non-RCTs. All the studies must be clinical study and the trials with cadaver and artificial models were excluded. Letters, comments, editorials, and practice guidelines were excluded. Cochrane Handbook for Systematic Reviews of Interventions was used to evaluate the methodological quality and risk bias of the included studies, the content of which includes method of randomization, allocation concealment, appropriateness of blinding, and whether the outcome data are complete. Two reviewers scanned the quality of the eligible studies independently. The quality of RCT studies was judged by using the Jadad 5-point scale and non-RCTs were judged by MINORS quality assessment. Discrepancies were resolved by consensus after discussion, and a third reviewer participated in the debate to determine the final outcomes if necessary.

### Data Extraction

Two reviewers independently read the titles and abstracts of the searched literatures. Most of the articles can be removed based on the topic of the article provided by their respective title or abstract and disagreements about whether or not included can be resolved by discussion. The postoperative pain intensity was measured by 100-point visual analogue scale (VAS). The 10-point VAS score and NRS score were converted by 100-point VAS.^8^ Data in other forms (ie, median, interquartile range, and mean ± 95% confidence interval [CI]) were converted to mean ± SD according to Cochrane Handbook.^9^ If the data were not reported numerically, we extracted them by Software “Getdata Graph Digitizer” from the published figures.

The following data were extracted and recorded in a sheet: demographic data about the patients in the literature, author's name, publication date, the sample size of the patients, location of study, the ratio of male and female, the dose and method that the ACB and FNB applied and whether it is the unilateral or bilateral TKA; the method of anesthesia; and the VAS score, opioid rescue consumption, time up and go test (TUG), the LOS, MVIC of quadriceps and adductor, the rates of complications(vomiting and nausea).

### Outcome Measures and Statistical Analysis

The main outcomes were the VAS score, opioid rescue consumption, TUG and the MVIC of quadriceps and adductor. Those main outcomes represent the effect of pain control and the strength of quadriceps. The second outcome was the LOS and the rates of complications. Continuous outcomes (VAS, TUG, the LOS, the strength of quadriceps and adductor) were expressed as the mean differences (MD) and respective 95% CIs. Discontinuous outcome (the rates of vomiting and nausea) was expressed as risk ratio (RR) with 95% CIs. Statistical significance was set at *P* < 0.05 to summarize findings across the trials, Software RevMan 5.3 (The Cochrane Collaboration, Oxford, United Kingdom) was used for meta-analysis and the software of Stata, version 12.0 (Stata Corp., College Station, TX) was used for sensitivity analysis. *I*^2^ were used to assess heterogeneity across studies, with *I*^2^ value of exceed 50% statistically heterogeneity. Sensitivity analysis was conducted to detected the stability of the consolidated results. Meta-analysis was performed using fixed effect or random-effect models according to the heterogeneity. When there was no statistical evidence of heterogeneity, a fixed effect model was adopted; otherwise, a random-effect model was chosen. Publication bias was tested using funnel plots.

## RESULTS

### Search Result

**FIGURE 2:**
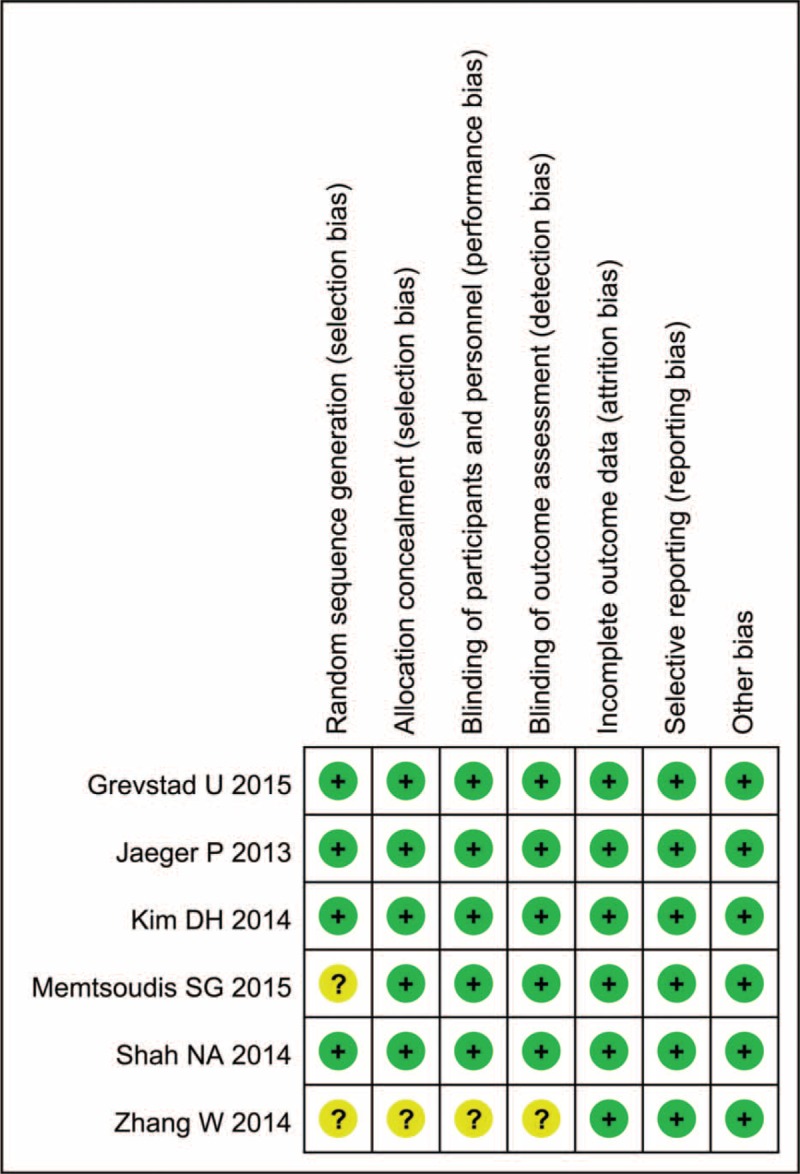
The result of the risk of bias summary.

In the initial search, we identified 422 potentially relevant studies, of which 80 duplicates were removed by Endnote Software. According to the inclusion criteria, 335 studies were excluded after reading the titles and abstracts. Finally, we included 8 clinical trials with 751 patients (751 knees) in the meta-analysis.^10–17^ Since on study performed the trial in one patient with one leg in ACB and another is in FNB.^11^ The characteristics of the studies that were included are shown in Table 1. Of the included studies, a total of 751 TKAs are performed and the number of ACB and FNB is 360 and 391, respectively; all articles were in English and published from the year of 2013. All participants in the 8 studies were the elderly that prepared for TKA. The mean age of the patients in the studies ranged from 61.9 to 70 years. The male patients and female patients are 98 and 243, respectively. The included 8 studies contained 6 RCTs and 2 non-RCTs published within 2 years. Only 2 trials^11,13^ did not state the random sequence generation and only 1 RCT^13^ did not state the allocation concealment, blinding of participant and personnel, and blinding of outcome assessment. The details of Cochrane Handbook for Systematic Reviews of Interventions can be seen in Figure 1 and Figure 2. Minor quality scores can be seen in Table 2.

**TABLE 1:**
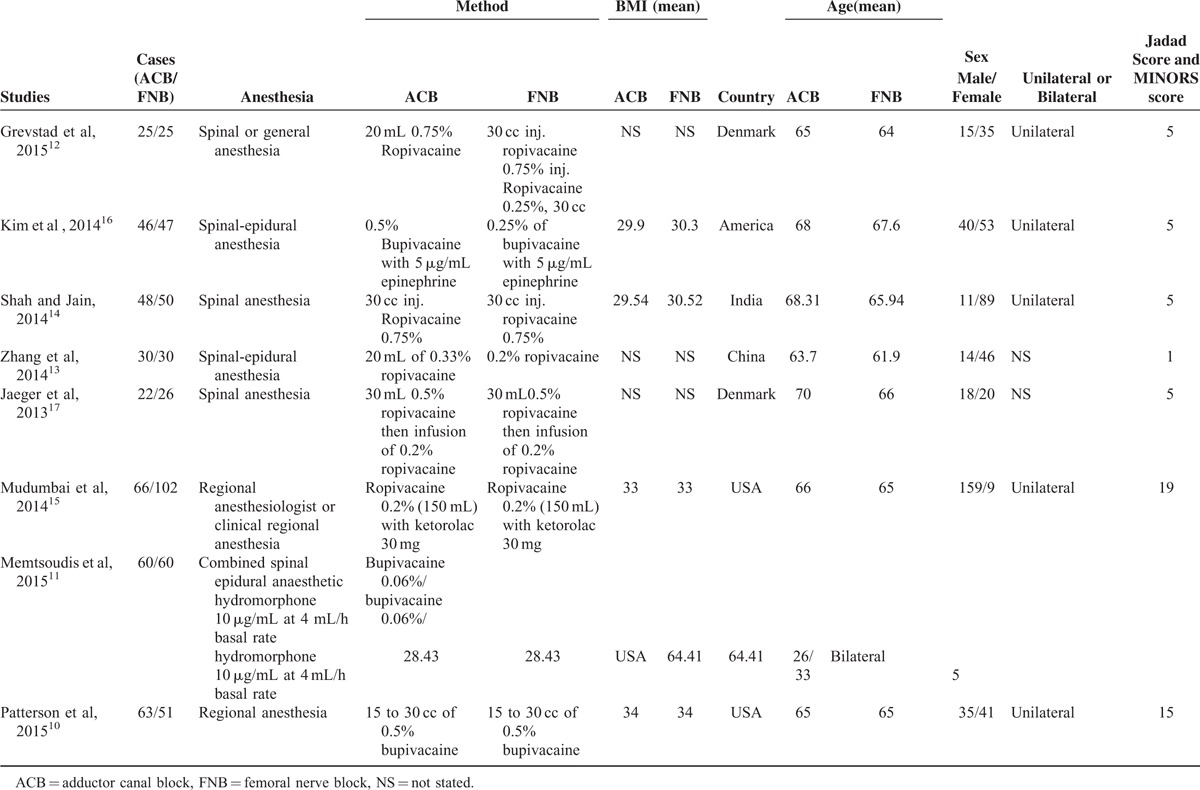
Characteristics of Included Studies

**TABLE 2:**
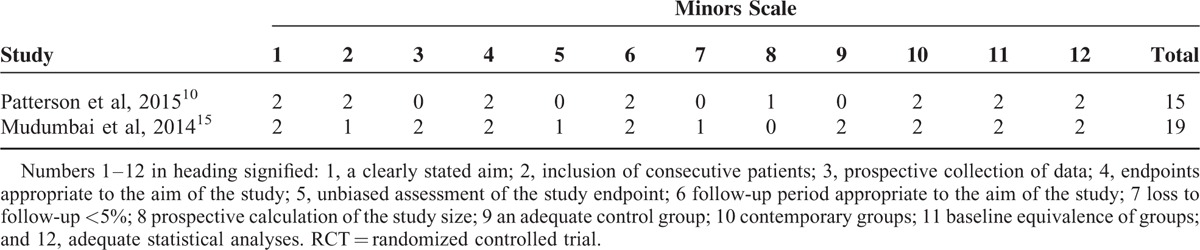
The Bias of the Non-RCTs Included in the Meta-analysis

### Results of Meta-analysis

#### VAS Score With Rest

**FIGURE 3:**
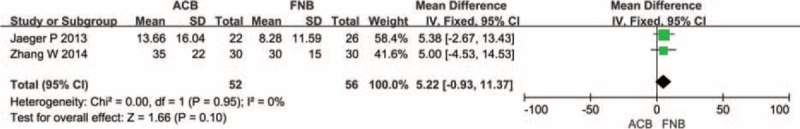
The meta-analysis of 2 trials included showed that there was no statistical significance between adductor canal block (ACB) and femoral nerve block (FNB) in terms of visual analogue scale score with rest at 4 h after total knee arthroplasty.

Only 2 studies with 108 TKAs showed the VAS score at 4 h postoperatively. Meta-analysis revealed no significant differences between the 2 groups (Figure 3) (MD = 5.22; 95% CI −0.93 to 11.37; *P* = 0.10).

**FIGURE 4:**
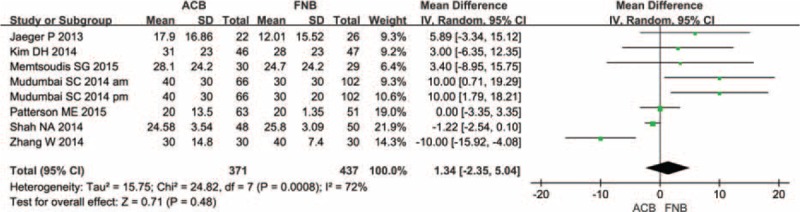
The meta-analysis of 4 trials included showed that there was no statistical significance between adductor canal block (ACB) and femoral nerve block (FNB) in terms of visual analogue scale score with rest at 24 h after total knee arthroplasty.

**FIGURE 5:**
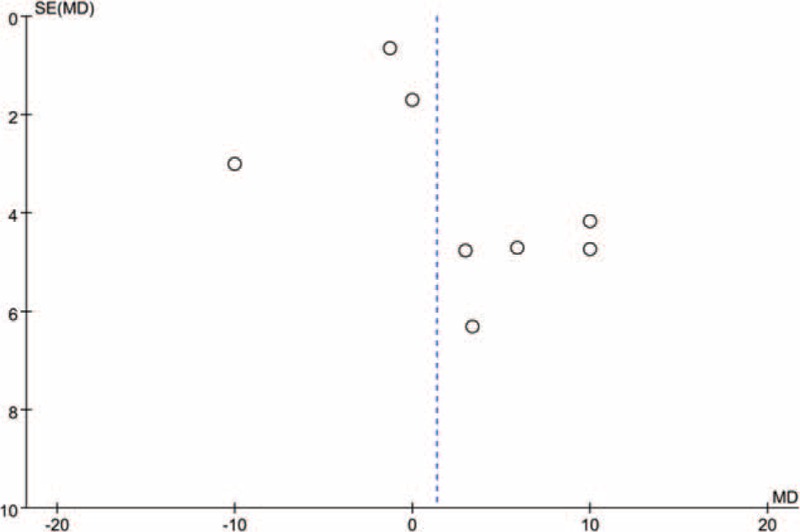
Funnel plot of studies analysing the effect of visual analogue scale score on the final results.

A total of 7 component studies (808 patients) provided VAS score at 24 h postoperatively. There was no statistically significant difference between the groups with respect to the VAS score at 24 h postoperatively (Figure 4) (MD = 1.34; 95% CI −2.35 to 5.04; *P* = 0.48). Studies included in Figure 4 were also assessed for any potential publication bias through a funnel plot (Figure 5). From the funnel plot, the horizontal axis meaning mean difference on and vertical axis stands for the standard error of the mean difference.

**FIGURE 6:**
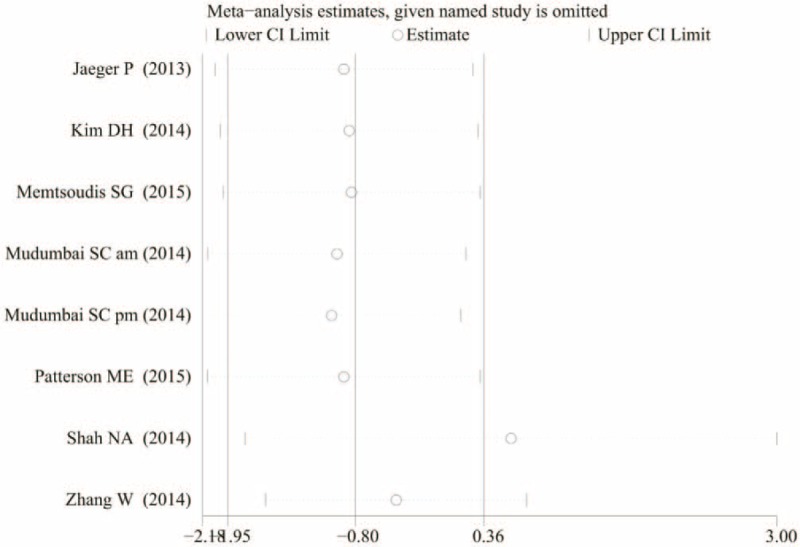
The sensitivity analysis of the visual analogue scale score at 24 h with rest.

To determine the source of heterogeneity and to enhance the credibility of our results, a sensitivity analysis was conducted. Based on the result of a sensitivity analysis, Shah and Jain showed a remarkable influence on heterogeneity (Figure 6). Shah and Jain's study administration 30 volume of 0.75% ropivacaine as deemed appropriate for ACB and this may the source of bias.

**FIGURE 7:**
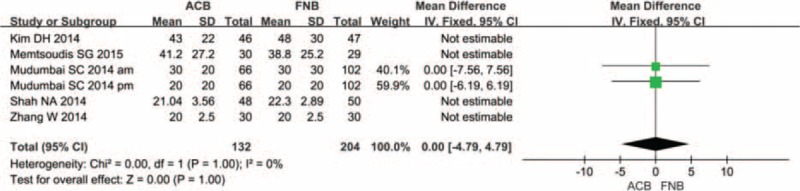
The meta-analysis of 2 trials included showed that there was no statistical significance between adductor canal block (ACB) and femoral nerve block (FNB) in terms of VAS score with rest at 48 h after total knee arthroplasty.

Only 5 studies with 646 TKAs showed the VAS score at 48 h postoperatively. Meta-analysis revealed no significant differences between the 2 groups (Figure 7) (MD = −0.62; 95% CI −1.50 to 0.25; *P* = 0.27).

### VAS Score With Mobilization

**FIGURE 8:**
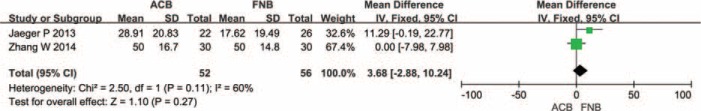
The meta-analysis of 2 trials included showed that there was no statistical significance between adductor canal block (ACB) and femoral nerve block (FNB) in terms of visual analogue scale score with mobilization at 4 h after total knee arthroplasty.

Two studies with 108 TKAs showed VAS score at 4 h postoperatively. No significant difference was found between the 2 groups (Figure 8) (MD = 3.68; 95% CI −2.88 to 10.24; *P* = 0.27).

**FIGURE 9:**
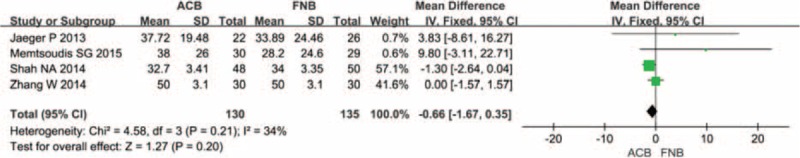
The meta-analysis of 2 trials included showed that there was no statistical significance between adductor canal block (ACB) and femoral nerve block (FNB) in terms of visual analogue scale score with mobilization at 24 h after total knee arthroplasty.

Up to 4 studies with 265 TKAs showed VAS score at 24 h postoperatively. No significant difference was found between the 2 groups (Figure 9) (MD = −0.66; 95% CI −1.67 to 0.35; *P* = 0.20).

**FIGURE 10:**
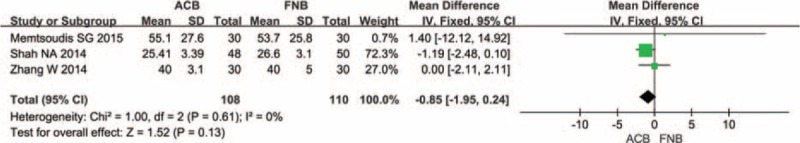
The meta-analysis of 2 trials included showed that there was no statistical significance between adductor canal block (ACB) and femoral nerve block (FNB) in terms of visual analogue scale score with mobilization at 48 h after total knee arthroplasty.

Only 3 studies with 218 TKAs showed the VAS score at 48 h postoperatively. Meta-analysis revealed no significant differences between the 2 groups (Figure 10) (MD = −0.85; 95% CI −1.95 to 0.23; *P* = 0.24).

### Opioid Rescue Consumption

**FIGURE 11:**
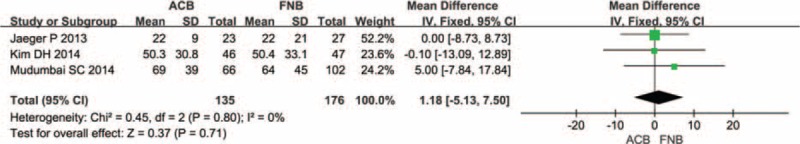
The meta-analysis of 3 trials included showed that there was no statistical significance between adductor canal block (ACB) and femoral nerve block (FNB) in terms of opioid rescue.

A total of 3 studies with 311 TKAs reported the outcomes of opioid rescue. Meta-analysis revealed no significant differences between the 2 groups (Figure 11) (MD = −1.18; 95% CI −5.13 to 7.50; *P* = 0.71).

### Length of Hospital Stay

**FIGURE 12:**
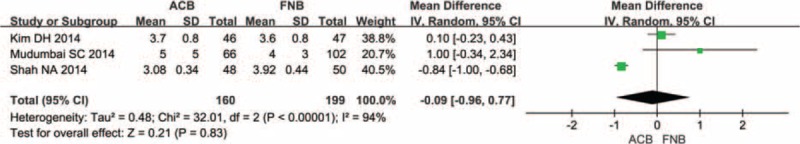
The meta-analysis of 2 trials included showed that there was no statistical significance between adductor canal block (ACB) and femoral nerve block (FNB) in terms of length of hospital stay.

A total of 3 component studies (359 patients) provided data on the LOS. The outcome of meta-analysis revealed that there is no significant difference between the 2 groups as referred to the LOS (Figure 12) (MD = −0.09; 95% CI −0.96 to 0.77; *P* = 0.83).

### MVIC of Quadriceps and Adductor

**FIGURE 13:**
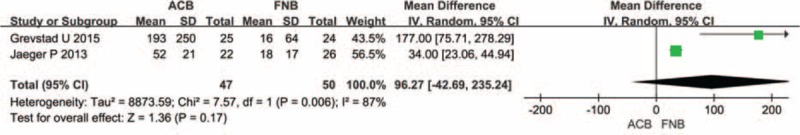
Forest plot diagram showing maximum voluntary isometric contraction of quadriceps of adductor canal block (ACB) and femoral nerve block (FNB) groups.

Two studies with 97 TKAs reported the MVIC of quadriceps postoperatively. Meta-analysis revealed that the 2 groups have no significant difference (Figure 13) (MD = 96.27; 95% CI −42.69 to 235.24; *P* = 0.17).

**FIGURE 14:**
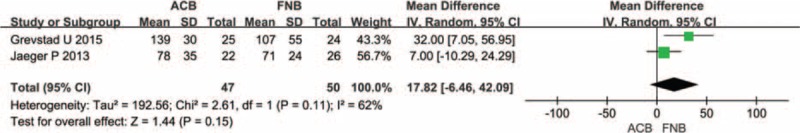
Forest plot diagram showing maximum voluntary isometric contraction of adductor of adductor canal block (ACB) and femoral nerve block (FNB) groups.

A total of 2 component studies (97 patients) provided data on the MVIC of adductor. The outcome of meta-analysis revealed that there is no significant difference between the 2 groups as referred to the MVIC of quadriceps (Figure 14) (MD = 17.82; 95% CI −6.46 to 42.09; *P* = 0.15).

### Complications

**FIGURE 15:**
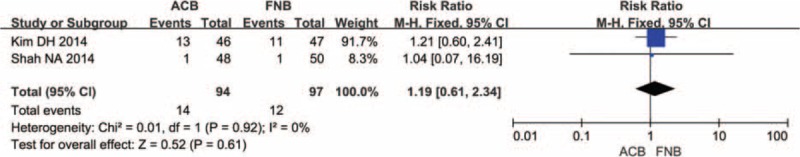
Forest plot diagram showing complication of adductor canal block (ACB) and femoral nerve block (FNB) on transfusion vomiting and nausea.

Two studies reported the data of complications. The results of meta-analysis showed that there was no statistical difference regarding nausea and vomiting (risk difference = 0.02; 95% CI = −0.07 to 0.12; *P* = 0.60) between the 2 groups (Figure 15).

## DISCUSSION

The present study is the first meta-analysis of RCTs and non-RCTs to compare the efficacy and safety of ACB and FNB in the management of pain after TKA. What's more, the most significant finding of this meta-analysis is that ACB shows same pain control effect than FNB; however, the strength of quadriceps and adductor has no significant difference. Finally, the LOS and complications between the 2 groups has no significant difference.

Pain degree was measured as VAS scores at 4, 24, and 48 h after TKA, and the results of combined analysis showed that the FNB group and ACB group have no significant difference of VAS score with rest or mobilization at 4, 24, and 48 h after TKA. Since the VAS score is a subjective scale and easy to be affected by the subjective factors. Subgroup analysis was conducted according to the RCTs and non-RCTs. The results indicated that there were no significant differences between the 2 groups in terms of VAS score at 24 and 48 with rest or mobilization (Table 3). This outcome concurs with the other studies of RCTs and non-RCTs in patients with TKA or healthy volunteers.^7,16–18^ The reason may be as follows: in the adductor canal, medial muscular ramus and the medial cutaneous ramus of the femoral nerve governing medial ligaments in addition to saphenous nerves.^19^ Additionally, the articular ramus of obturatorius nerves enters the adductor canal at the distal end.^20^ Finally, the most nerves in the adductor canal are sensory nerves governing knee joints. However, Perlas et al^21^ investigated the analgesia effect of local infiltration and ACB, it shows that LIA plus ACB show better analgesia outcome than FNB after TKA, whereas, it is not clear that which is the more effective method to control pain after TKA. Many letters have concerned the Shah and Jain,^14^ their main concerns are the timing and volume of the block, which contain 30 mL of 0.75% ropivacaine followed by 30 mL of 0.25% every 4 h. The fact is that a previous MRI based study on ACB considered 30 mL of volume as deemed appropriate for ACB.^22^ In addition, the volume and concentration in ACB and FNB are just equal to each other; moreover, they fitted into our inclusion criteria, and after careful consideration, the final decision is that it should be included in our meta-analysis. In addition to this, a study has performed the study in bilateral knee arthroplasty, this may affect the outcome, since bilateral will cause more pain than unilateral knee arthroplasty.^11^

**TABLE 3:**
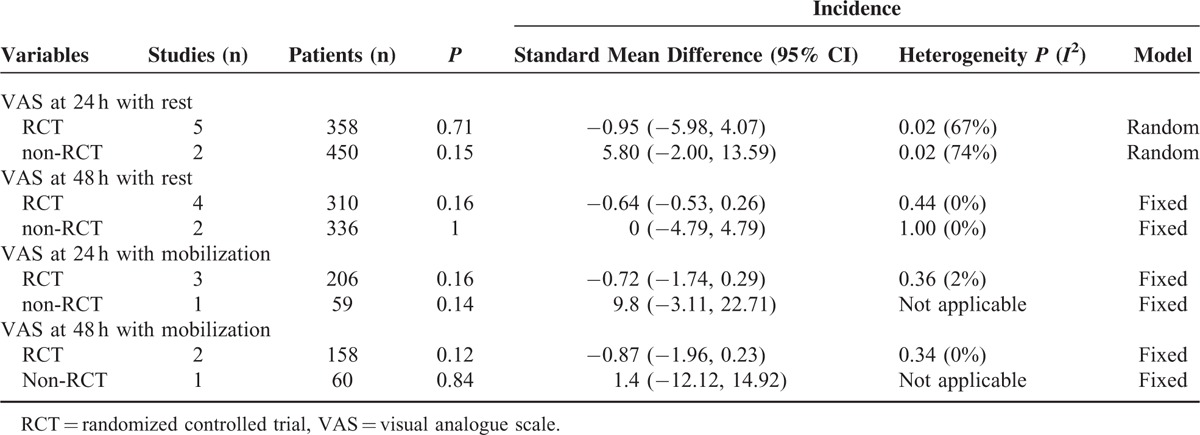
The Subgroup Analysis of the VAS Score at 24 and 48 h With Rest or Mobilization

However, this study only estimates the VAS score with mobilization at 24 and 48 h, and the result did not show the heterogeneity. Since spinal anesthesia and general anesthesia will cause different analgesia effect on the TKA, however, included study in general analgesia or spinal analgesia ^12^ did not measure the VAS score with rest or mobilization 4 h after TKA. In our meta-analysis, this will not influence the final decision. In addition to this, timing and volume of the block also different from each other, the most controversial study is the Shah and Jain's study, in their opinion, 30 mL of 0.75% ropivacaine can be filled the adductor aponeurotic space. Thus, even the posterior branch of abturator nerve joins the canal can be blocked with this volume local anesthetic^22^. Different dose and timing may have influence on the final result; in my opinion, this will have a little impact on the result. Since it has been established that even an small volume and low concentrations of local anesthetics have an immense effect on quadriceps.^6^ The optimum dose for control pain is still needed to explore in the subsequent study.

As referred to quadriceps strength, which can improve physical therapy for TKA, is important for relieve pain for TKA patients. Previous studies show that ACB preserves quadriceps strength and enhance ambulation compared with FNB.^7,15–17,23^ However, our meta-analysis revealed that ACB showed no better postoperative outcomes with regard to ambulation ability and muscle strength of adductor and quadriceps in comparison to FNB post TKA. The muscle strength was measured by MVIC and ambulation ability was judged by TUG test. Since the other data that measured the muscle strength cannot be got or only one study measured, we only perform the MVIC of quadriceps and adductor to assess the muscle strength postoperative. The outcome is not consistent with the present outcomes of other studies. Theoretically since all nerves that traverse the adductor canal are only sensory nerves, ACB would have less impact on quadriceps strength, while relieving pain. So, this outcome should be cautiously treated; since the sample is not enough, the dose of the analgesia is different and the criteria that measured muscle strength are different.

As referred to complications and LOS, an ideal method of analgesia is to relieve pain while not increasing the fatal complication.^24^ The LOS represents the economic expend of each patient. This meta-analysis indicated that ACB and FNB have no significant difference in form of these 2 respects. Since they both can control the pain of TKA, the consumption of opioid and their related complications will decrease. Both of the 2 methods can control pain effectively, so the patients will shorten the hospital stay. However, the LOS is dependent not only on efficacy of pain control, but also the recovery of the patients. In our meta-analysis, there is large heterogeneity between the 2 groups, so the outcomes should be treated cautiously. Besides these, peripheral nerve blocks may relate to few noteworthy complications like nerve injury, catheter site infection, and heel ulcers.^25^

This meta-analysis have some potential limitations. First, only 6 RCTs and 2 non-RCTs compared the ACB and FNB in the pain management after TKA and the sample is not enough. Thus, more high-quality RCTs are still needed to identify the most effective pain control method. Second, the low Jadad score RCT and 2 non-RCTs will have impact on the final outcomes. Besides these, a study compares the ACB and FNB in pain management in bilateral TKA; this will have influence on the final result. Third, the meta-analysis has some invalid data such as time to achieve straight leg raise. Fourth, the publication bias existed in all meta-analyses can also affect the result. Fifth, the different anesthesia including spinal, general, or spinal-epidural was used in the included studies, which will cause great clinical heterogeneity; otherwise, the dose of different drug administration in ACB and FNB will also have an influence on the final conclusion. Lastly, many other analgesia methods were used in different trials, which may produce some bias too.

## CONCLUSION

The present meta-analysis indicated that the ACB and FNB have no significant difference in forms of VAS score with rest of mobilization at 4, 24, 48 h, the muscle strength of adductor and quadriceps, the LOS, and complications.
